# Pilot Date on Swallow Function in Nondysphagic Patients Requiring a Tracheotomy Tube

**DOI:** 10.1155/2009/610849

**Published:** 2009-10-26

**Authors:** Susan L. Brady, Michele Wesling, Joseph Donzelli

**Affiliations:** ^1^Department of Speech-Language Pathology, Marianjoy Rehabilitation Hospital, 26 West 171 Roosevelt Road, Wheaton, IL 60187, USA; ^2^Department of Otolaryngology, Marianjoy Rehabilitation Hospital, 26 West 171 Roosevelt Road, Wheaton, IL 60187, USA; ^3^MidWest Ears, Nose, & Throat, 1247 Rickert Drive no., Naperville, IL 60540, USA

## Abstract

*Objective*. To evaluate the effects of occlusion status (i.e., open, finger, capped) of the tracheotomy tube and removal of the tracheotomy tube that may have upon bolus flow and durational measurements in nondysphagic persons requiring a tracheotomy tube. *Study Design*. Prospective, single subject, repeated measure design. *Methods*. Participants had their swallow evaluated with 5 mL pureed boluses using nasal endoscopy with the tracheotomy tube in place, removed, and under the following occlusion conditions: open, finger, and capped. The order of occlusion condition was randomized. *Results*. Aspiration was never observed but laryngeal penetration was a common finding. Durational measurements for swallow initiation and duration of white out were not significantly different by occlusion status or after removal of the tracheotomy tube. *Conclusion*. This study provides corroborating evidence demonstrating the lack of a relationship between a tracheotomy tube and swallowing dysfunction.

## 1. Introduction

It is essential for dysphagia rehabilitation clinicians to understand the complex nature of swallowing in nondysphagic persons with a tracheotomy in order to effectively treat patients with a tracheotomy who have a compromised swallow. There is debate in the literature about the role and influence the presence of a tracheotomy tube and occlusion status may have upon a person's ability to swallow [1–13]. Even though the basic pharyngeal biomechanics of the swallow have been previously studied in individuals with a tracheotomy tube [1–12], much of this research involved only patients with a known or suspected dysphagia. The current lack of research regarding swallowing ability in patients with a tracheotomy tube without any known or suspected dysphagia leaves clinicians with the challenge of parceling out the influence of the underlying neurologic and/or medical condition that necessitated the tracheotomy tube in the first place from the presence of the tracheotomy tube alone [10–12].

Swallowing function can be evaluated by either videofluoroscopy swallowing study (VFSS) or fiberoptic endoscopic examination of the swallow (FEES). Both instrumental assessments have demonstrated good agreement for the detection of swallowing abnormality and can be used to compliment each other [14]. Terk, Leder, and Burrell [13] were the first to use objective means to investigate the effects of the presence of the tracheotomy tube and occlusion status may have upon specific pharyngeal biomechanical aspects of the swallow using videofluoroscopy. They found that the presence of a tracheotomy tube did not significantly alter hyoid bone movement or laryngeal excursion when the tracheotomy tube was removed and under various occlusion conditions. This was the first study to provide objective evidence regarding the pharyngeal biomechanics of the swallow function in patients with a tracheotomy and normal swallow function.

Research with nondysphagic patients who require a tracheotomy tube is important as it can potentially improve understanding the relationship between the presences of a tracheotomy tube and swallowing function. The purposes of this research project were to evaluate what effect, if any, do different occlusion conditions of the tracheotomy tube (i.e., open, finger, capped) and removal of the tracheotomy tube have upon bolus flow and durational measurements for a pureed bolus as viewed by nasal endoscopy.

## 2. Materials and Method

### 2.1. Subjects

Participants for this study were recruited from a referred population of patients who were evaluated by otolaryngology for management of their tracheotomy tube. Inclusion criteria included subjects with no reported swallowing problems, consuming a regular diet, had normal cognition, and could tolerate decannulation for a short period of time. All subjects signed an informed consent statement. This facility's institutional review board approved this study.

### 2.2. Procedure

The study participants had their swallow evaluated by FEES using established clinical protocols [15–17]. The flexible nasal laryngoscope used was the Olympus ENFP3 and was placed in the high position before and during the swallow and then advanced to the low position after the swallow. All examinations were recorded and durational measurements were obtained using the Kay Advanced Digital Endoscopy/Stroboscopy system version 3.31. The light source used was the Kay RLS 9100B Rhino-Laryngeal Stroboscope (Lincoln Park, NJ). No topical anesthesia was used during the study. All tracheotomy tube cuffs were deflated. Tracheotomy tube occlusion conditions, that is, open, finger, and capped, were randomized. The tracheotomy tube out condition was evaluated last by placing a 4 × 4 gauze sponge gently over the tracheostoma site.

Each puree bolus (applesauce) was measured with a syringe and presented on a spoon to each participant in a 5-mL bolus volume. The subjects were instructed to hold the bolus in their mouth until given the command to swallow. Prior to each swallow, the subjects were informed what condition they were swallowing under.

### 2.3. Data Analyses and Interjudge Reliability

Two speech language pathologists independently evaluated each swallow for bolus flow and durational measurements. Bolus flow was evaluated for laryngeal penetration and aspiration and also using the Penetration-Aspiration Scale (PAS) developed by Rosenbek and colleagues [18]. The PAS is an eight-point scale that measures the depth and response to airway invasion of the bolus. The higher the score, the deeper the bolus invaded the airway where a 1 = no material entering the airway and an 8 = material entering the airway below the level of the vocal folds, is not ejected, and does not respond to the air invasion (silent aspiration). Levels 1–5 on the PAS reflect different degrees of laryngeal penetration and levels 6–8 reflect different degrees of aspiration. Interjudge reliability for laryngeal penetration and aspiration using the kappa correlation coefficient between the two raters for the presence or absence of penetration and aspiration was .856 (*P* = .0001) which reflects very good agreement.

The two durational measurements calculated were the swallow initiation time (SIT) and the complete “white-out” time (WOT). Each examination was reviewed in a frame-by-frame analysis. The recording equipment used for this study allowed for durational analyses which allow the investigator to mark the examination film at specific points using a running frame-by-frame counter with a capture film rate of 30 frames per second. SIT was defined as the time from when the bolus head reached the horizontal level at the tip of the epiglottis to the start of the complete “white out.” If the bolus head was not viewed prior to the “white-out” a score of 0 seconds was assigned to that swallow. The complete WOT was defined as the total amount of time that the entire view screen was completely “white.” The “white out” was considered to include the time of complete laryngeal closure. The Pearson product moment correlation between the two raters for durational measurements was .97 (*P* ≥ .001) which reflects very good agreement. Therefore, only the first rater's measurements were used for further analyses.

Statistical analyses were completed using SPSS version 14.0 computer software program. ANOVAs were completed on the continuous variables for the durational measurements and the penetration-aspiration scale score. The alpha level for all tests was set at 0.05 level of significance.

## 3. Results

### 3.1. Subjects

A total of six subjects (3 M/3F; age range 34–88 years, mean age 64.4 years ± 24.32 years) participated in this pilot study. Time from tracheotomy until FEES ranged from 46 to 252 days with a mean of 118 days ( ± 89.6 days). Additional demographics are summarized in Table 1.

### 3.2. Aspiration/Laryngeal Penetration/Pharyngeal Residue

None of the subjects demonstrated tracheal aspiration during any of the swallowing tasks. Five out of the 6 subjects demonstrated laryngeal penetration under one or more of the testing conditions. Table 2 summarizes the testing conditions when laryngeal penetration was present. No optimal swallowing patterns were identified under the various conditions. Laryngeal penetration was present when the tracheotomy tube occlusion status open with only one of the subjects, finger occlusion with four of the subjects, capped with two of the subjects, and with the tracheotomy tube removed with 3 of the subjects. Subject number three demonstrated laryngeal penetration under all of the testing conditions.

When the results were evaluated by the mean PAS level for each testing condition across the six subjects, no significant differences were observed. All PAS levels rated were from level 1 (material does not enter the airway) to level 3 (material enters the airway, remains above the vocal folds, and is not ejected from the airway). None of the swallows were rated greater than a PAS level 3. Table 3 provides specific mean PAS level for each occlusion condition.

### 3.3. Durational Measurements

Overall, durational measurements for swallow initiation time ranged from 0–5.5 seconds with a mean of 0.54 seconds (SD = 1.09 seconds). The differences between the mean SIT times under the various occlusion conditions were not significant (F = 0.61; *P *= .62). Overall, the duration of “white-out” time ranged from 0.13–1.8 seconds with a mean of 0.63 seconds (SD = 0.19 seconds). The differences between the mean WOT under the various occlusion conditions were not significant (*F* = 1.24, *P* = .33). Table 4 summarizes the mean durational times for SIT and WOT for each occlusion condition.

## 4. Discussion

This paper presents corroborating evidence derived from a prospective study regarding the bolus flow and durational measurements of the swallow using FEES in nondysphagic persons requiring a tracheotomy tube. The results of this pilot study revealed that aspiration was never observed but laryngeal penetration was a common finding. Durational measurements for swallow initiation and duration of “white out” were not significantly different by occlusion status or after removal of the tracheotomy tube.

This study finding concurs with the previous research which demonstrated that laryngeal penetration is present in nondysphagic individuals without a tracheotomy tube [19–21]. Even though minimal changes were observed with swallowing function for bolus flow with increased level of penetration observed, no clear pattern for optimal swallowing condition was identified. The durational analysis for swallow initiation time and duration of “white out” was not influenced by occlusion status or the presence of the tracheotomy tube. This finding provides additional evidence of the limited relationship between swallow function and the presence of a tracheotomy tube [10–12].

Limitations of this study were the small sample size (*N* = 6) and the use of only puree boluses. This restriction was required for the investigation by the institutional review board since at the time of the conceptualization of this research project, the risk of the proposed research project was essentially unknown. Since the results showed no increased risk for aspiration under the various swallowing conditions, direction for future research includes replicating this study using liquids and with a larger sample size.

## 5. Conclusion

Understanding the nature of swallowing in persons with a tracheotomy tube without swallowing difficulties is essential to understand swallowing difficulties in patients with a tracheotomy tube and dysphagia. This study provides corroborative evidence to the growing body of literature demonstrating the lack of a relationship between the presences of a tracheotomy tube and swallowing dysfunction [10–12].

Laryngeal penetration in patients with a tracheotomy tube without dysphagia is a common finding. No optimal tracheotomy tube occlusion condition was indentified to eliminate the laryngeal penetration. Duration of swallow initiation time and complete “white out” was not significantly different by occlusion status or after the removal of the tracheotomy tube.

## Figures and Tables

**Table 1:** Subject demographics.

Subject	Gender	Age (years)	Diagnosis	Duration of tracheotomy prior to FEES	Type of tracheotomy tube
1	F	88	Respiratory failure secondary to COPD	59 days	Bivona no. 5, noncuff
2	M	34	Sleep apnea and COPD	207 days	Shiley no. 9, cuffed
3	M	77	Sleep apnea and COPD	252 days	Bivona no. 5, noncuff
4	M	36	Respiratory failure secondary to incomplete SCI at level of C5-C6	46 days	Shiley no. 7, noncuff
5	F	63	Respiratory failure secondary to blastomycosis	98 days	Bivona no. 6, noncuff
6	F	87	Respiratory failure status post cardiac surgery	46 days	Shiley no. 6, cuffed

**Table 2:** Penetration/aspiration.

Subject	Penetration-aspiration scale	Occlusion condition
1	3	Finger
		No tracheotomy tube
3	3	Open
		Finger
		Capped
		No tracheotomy tube
4	2	Finger
5	2	No tracheotomy tube
6	2	Finger
		Capped

**Table 3:** Mean penetration-aspiration level by occlusion condition.

Occlusion condition	Mean penetration/aspiration level
Open	1.44 (SD = 0.88)
Finger	1.30 (SD = 0.67)
Capped	1.33 (SD = 0.82)
No tracheotomy tube	1.63 (SD = 0.916)
Nonsignificant (F = 0.262, *P *= 0.852)	

**Table 4:** Durational measurements by occlusion condition.

Occlusion Condition	Mean swallow initiation time(seconds)	Mean “white-out” time (seconds)
Open	0.483 (SD = 0.425)	0.661 (SD = 0.343)
Finger	0.726 (SD = 1.20)	0.711 (SD = 0.098)
Capped	1.319 (SD = 2.34)	0.726 (SD = 0.086)
No tracheotomy tube	0.279 (SD = 0.216)	0.506 (SD = 0.208)
